# Using single-dose liposomal amphotericin B for cryptococcal meningitis induction therapy: nurse pearls and practical perspectives

**DOI:** 10.12688/wellcomeopenres.21450.1

**Published:** 2024-05-15

**Authors:** Jane Frances Ndyetukira, Richard Kwizera, Cynthia Ahimbisibwe, Carol Namujju, David B. Meya

**Affiliations:** 1 Department of Research, Infectious Diseases Institute, College of Health Sciences, Makerere University, Mulago, Kampala, Uganda; 2 Department of Medicine, School of Medicine, College of Health Sciences, Makerere University, Mulago, Kampala, Uganda; 3 Division of Infectious Diseases and International Medicine, Department of Medicine, University of Minnesota Twin Cities, Minneapolis, Minnesota, USA

**Keywords:** Liposomal amphotericin B, Cryptococcal meningitis, Research nurse, Clinical research

## Abstract

**Background:**

In Uganda where the burden of HIV-associated cryptococcal meningitis is high, conventional amphotericin B deoxycholate has been standard to manage patients with cryptococcal meningitis in research settings. However, liposomal amphotericin B (AmBisome) is now available via the efforts of UNITAID. We sought to describe our nursing experience using AmBisome within a clinical trial for cryptococcal meningitis.

**Methods:**

We describe the experience of using single-dose 10mg/kg liposomal amphotericin B from the perspective of a research nurse in Uganda. Second, we described the process of preparing and administering amphotericin. Third, we assessed the nursing time required for the administration of daily amphotericin B versus single-dose liposomal amphotericin. Fourth, we discuss the major challenges faced while using liposomal amphotericin B.

**Results:**

We provide estimates for the nursing time required for reconstituting, filtering, diluting and administering liposomal amphotericin B and a visual aid for nursing tasks. Based on five trained nurses, the process of reconstitution and filtration lasts an average of 52 minutes (Range: 40 to 60 minutes), to reconstitute a mean of 11 (range: 8 to 15) 50mg vials (median weight 55kg). Overall, less nursing time was required for single-dose administration than for daily amphotericin B dosing. From a nursing perspective, liposomal amphotericin B was preferable to amphotericin B deoxycholate due to its reduced infusion reactions and other toxicities.

**Conclusions:**

Single-dose liposomal amphotericin B is a better alternative to daily amphotericin B. In addition to less toxicity, nosocomial infections, reduced hospital stay, and the potential for lower hospitalisation costs, the nursing implications should not be discounted. Quality nursing care is a finite resource in low- and middle-income countries, and single-dose amphotericin B reduced the nursing time required for the care of patients with cryptococcal meningitis.

## Plain language summary

In Uganda where the burden of HIV-associated cryptococcal meningitis is high, conventional amphotericin B deoxycholate has been standard to manage patients with cryptococcal meningitis in research settings. However, liposomal amphotericin B is now available via the efforts of UNITAID. Liposomal amphotericin B is known to be less nephrotoxic than amphotericin B deoxycholate. We demonstrated that liposomal amphotericin B is a better alternative to amphotericin B deoxycholate with less toxicity, nosocomial infections, reduced hospital stay, and the potential for lower costs of hospitalisation for both the patient and the healthcare system given the single, 10 mg/kg regimen. Despite the perceived high cost of liposomal amphotericin B and the tedious reconstitution process requiring training and adequate manpower, we believe that single-dose liposomal amphotericin B has enough advantages over the deoxycholate formulation to compel Ministries of Health to consider procuring liposomal amphotericin B as the drug of choice for the management of HIV-associated cryptococcal meningitis. The rollout will require training to facilitate widespread implementation, and we hope that this paper will serve to facilitate this training for nurses who will be using liposomal amphotericin B.

## Introduction

HIV-associated cryptococcal meningitis accounts for 15–19% of AIDS-related deaths ^1– 5^. About 9% of Ugandans are affected by serious fungal diseases annually with an annual incidence of cryptococcal meningitis estimated at 5553 cases ^6^. Before June 2022 ^7^, the World Health Organization (WHO) guidelines for cryptococcal meningitis recommend induction therapy with seven days of intravenous (IV) amphotericin B deoxycholate and oral flucytosine followed by seven days of oral fluconazole ^8^. However, in resource-limited settings where flucytosine is not always available, 14 days of amphotericin B deoxycholate and oral fluconazole 1200mg/day is recommended. In this scenario, treatment with amphotericin B deoxycholate requires 14 days of intravenous infusions given in hospital settings. Amphotericin B deoxycholate also causes many side effects including phlebitis, nausea, vomiting, rigors, anaemia, nephrotoxicity, and electrolyte abnormalities ^9^. These necessitate regular laboratory monitoring and additional medication management, making amphotericin difficult and costly to administer as there are additional healthcare system costs beyond the medication alone. As a result, high-quality nursing care is a critical component in managing these patients ^10^. Besides, recent studies showed that guidelines are generally not being adhered to by health workers while managing cryptococcal meningitis in Uganda ^11^.

In Uganda where the burden of cryptococcal meningitis is high ^1^, conventional amphotericin B deoxycholate has been standard to manage patients with cryptococcal meningitis in research settings ^12– 14^. However, a modified formulation of amphotericin B, liposomal amphotericin B (AmBisome, Gilead Sciences, Foster City, CA, USA) is now available via efforts of UNITAID ^15^. Liposomal amphotericin B has historically been mainly available in high-income countries. The liposomal formulation is considerably less toxic than the conventional amphotericin B deoxycholate ^16^.

In this paper, we aimed to describe the experience of using single-dose liposomal amphotericin B from the perspective of a research nurse in a resource-limited setting. Second, we described the process of preparing and administering amphotericin. Third, we assessed the nursing time required for the administration of daily amphotericin versus single-dose liposomal amphotericin. Fourth, we discuss the major challenges faced while using liposomal amphotericin B.

## Methods

### Study design and setting

This was an observational study nested under the AMBITION-cm clinical trial. In October 2018, the Infectious Diseases Institute of Makerere University began recruiting participants in Kampala, Uganda as one of the sites of the AMBIsome Therapy Induction OptimisatioN (AMBITION-cm) trial: ( ISRCTN72509687) ^17^. This was a Phase III multinational Randomised Controlled Non-Inferiority Trial that aimed to determine whether a single 10mg/kg dose of liposomal amphotericin was as effective as the standard treatment in terms of preventing deaths from cryptococcal meningitis. Participants were recruited from Mulago National Referral Hospital, Kampala, and Mbarara Regional Referral Hospital, Mbarara, Uganda. The AMBITION-cm trial found that a single, high-dose of AmBisome given with flucytosine and fluconazole was non-inferior to the current WHO-recommended standard of care in averting all-cause mortality at 10 weeks ^17^. The AmBisome regimen effectively cleared cryptococcus from the CSF and was associated with a significant reduction in adverse events. The reduced adverse events in turn reduced the care burden on the side of nurses. This regimen offers a practical, easier-to-administer and better-tolerated treatment for HIV-associated cryptococcal meningitis in Africa, with the potential to reduce the length of hospital admissions. This was the first time we were introduced to the use of liposomal amphotericin B in Uganda.

### Study participants

Participants included in the AMBITION-cm trial were HIV-positive adults (age ≥ 18 years) who had a first episode of cryptococcal meningitis, as diagnosed on the basis of a positive India ink stain or cryptococcal antigen test (CrAg lateral flow assay, IMMY) of a cerebrospinal fluid sample. Participants were excluded if they had received more than two doses of either amphotericin (at any dose) or fluconazole (at a dose of ≥800 mg) before screening; declined to consent or, if they had impaired capacity to consent, had no legal representative to consent on their behalf; were pregnant or breast-feeding; were taking contraindicated concomitant drugs; or had had any previous adverse reaction to a trial drug.

### Ethical consideration

The study was conducted in compliance with the approved protocol, the Declaration of Helsinki 2008, the principles of Good Clinical Practice and applicable national regulations. The protocol was approved by the London School of Hygiene and Tropical Medicine Research Ethics Committee (on 09/06/2017, ref: 14322) and Mulago Hospital Research and Ethics Committee (MHREC 1297). All the participants provided written informed consent.

## Results

### Reconstitution and filtration of AmBisome

**Figure 1.**
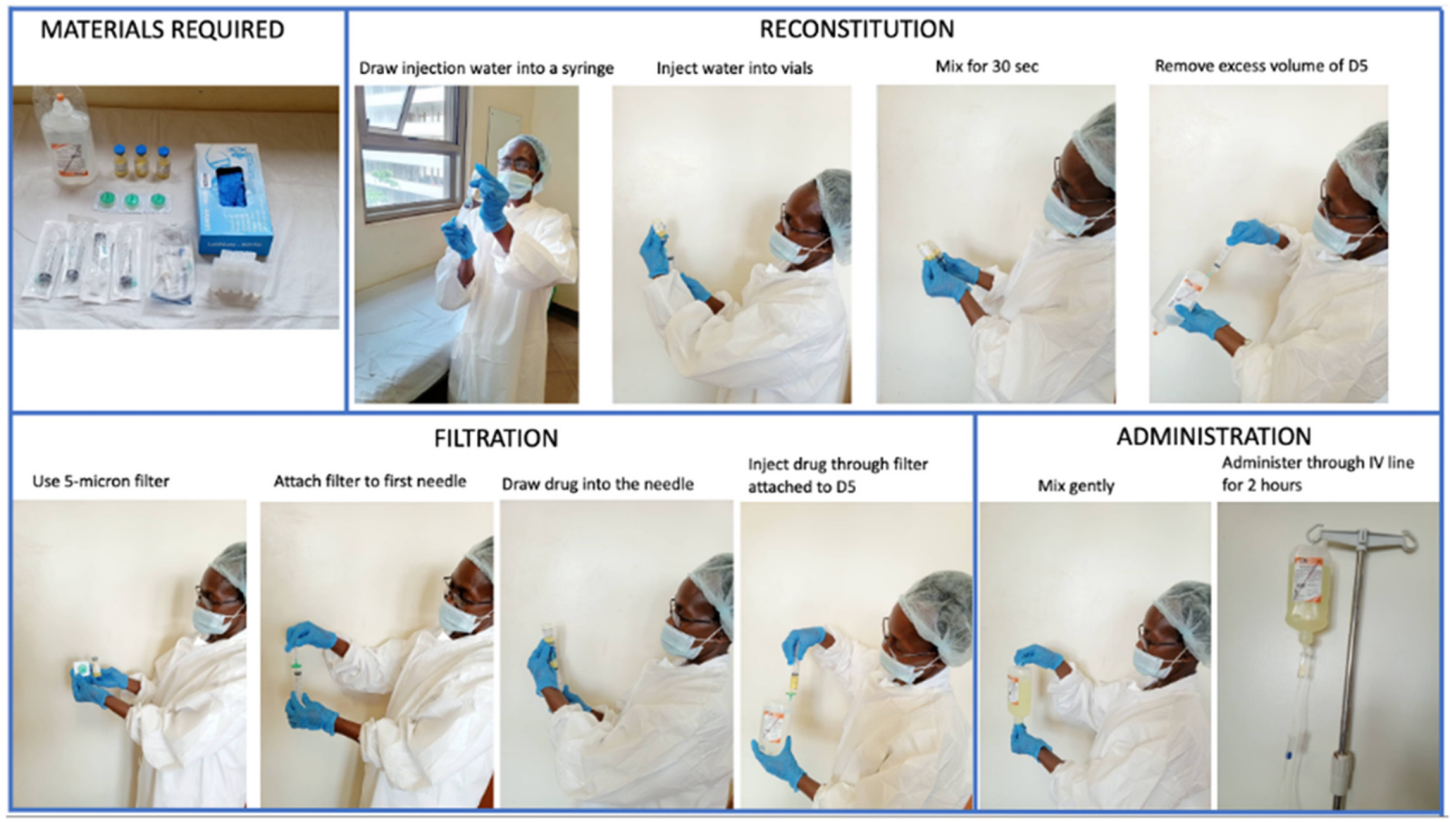
Steps in reconstitution of AmBisome. The whole process of reconstitution and filtration lasts an average of 52 minutes to reconstitute an average of 11 vials.

Before reconstitution, patients receive one liter of IV fluids (Normal saline or Ringer’s lactate) and 13meq of IV potassium chloride injected into the first bottle (500 ml) to run over two hours. Care must be taken not to exceed the safe rate of potassium infusion. Drug preparation for AmBisome (50 mg) involved reconstituting the powder with sterile water followed by filtering using a provided sterile 5-micron filter before mixing into 5% dextrose. Depending on the patient’s weight, an average of 11 vials of 50mg AmBisome were reconstituted to make one dose (10mg/kg/body weight) for each patient living with HIV in Uganda. Reconstitution of the drug was time-consuming as it required several steps. Briefly, the procedure would require 20ml syringes with, water for injection, filters, 2 bottles of 5% dextrose (500 ml), sterile gloves, and worksheets. This necessitated calculating the dose and therefore the number of drug vials based on the patient’s body weight. To reconstitute, the healthcare worker would don sterile gloves, draw 12 ml of water for injection into the drug vial and shake the vial for at least 30 seconds, and eventually draw the drug into the syringe aseptically. The drug would then be transferred into the bottle through a 5-micron filter with the cannula *in situ* ( Figure 1).

### Quantification of nursing time

Based on the five nurses we trained and timed, the whole process of reconstitution and filtration lasts between 40 to 60 minutes with an average of 52 minutes, to reconstitute between 8 to 15 (average 11) vials. This is longer than would be required to reconstitute amphotericin B deoxycholate (~5min); however, this is only done once as opposed to being repeated daily for 7–14 days with traditional amphotericin B dosing. The reconstitution had to be done under aseptic conditions since many syringes were required.

### Drug administration

After reconstitution, the amount of injection water used to reconstitute AmBisome must be removed from the 500 ml of 5% dextrose before adding in the reconstituted drug. Before connecting the drug to the vein, it is flushed with 10–20ml of water for injection before and after the drug. It is then set to run at a rate of 83 drops/min (~two hours). This is followed by 500 ml of 5% dextrose and one liter of normal saline or Ringers’ lactate as post-load. As given in the trial and recommended by WHO ^7^, oral magnesium and potassium tablets were given for three days. AmBisome takes two hours to administer, a shorter time compared to amphotericin B deoxycholate which generally takes four hours. Increasing the rate of deoxycholate infusion increases the risk of infusion reactions.

### Experience with AmBisome

As compared to amphotericin B deoxycholate which is available in several public and private pharmacies, all the health workers at our site had no prior experience of using AmBisome due to its previous unavailability. We had a series of training sessions on how to prepare and administer the drug. Unopened vials of AmBisome require storage below 25°C (~ambient temperature) dispensing of the need for cold chain maintenance compared to amphotericin B deoxycholate, which needs refrigeration (2°-8°C) until reconstitution. The reconstituted AmBisome product concentrate may also be stored for up to 6 hours at 2°- 8°C following reconstitution with sterile water for injection. Best practices would require continuous temperature monitoring and daily recording of min/max temperatures. This is challenging in our setting where backup power supply systems and refrigerators may be unavailable.

On the other hand, we clinically observed fewer drug reactions like rigors, anaemia (Grade 3 or 4, 13% in the AmBisome arm vs 41% in the control), need for blood transfusion (8% vs 18%), hypokalaemia (grade 3 or 4, 2% vs 7%), thrombophlebitis requiring antibiotic therapy (2% vs 7%) and vomiting among patients on AmBisome as compared to those with amphotericin B deoxycholate ^17^. Similarly, patients on AmBisome seemed to clinically improve quickly compared to those receiving amphotericin B deoxycholate, probably due to less toxicity and similar efficacy. AmBisome being a single dose, there is less intravenous cannulation that can contribute to thrombophlebitis and bacteraemia risk compared to amphotericin B deoxycholate, which needs frequent changing of intravenous sites to avoid phlebitis ^18, 19^.

## Discussion

### Challenges

The reconstitution process required more sundries for a single patient, which might be problematic in a routine clinic setting. Related to the above, in most low-and-middle-income settings, there is a challenge of understaffing. Using single-dose AmBisome will be a solution to the manpower problem as the few nurses would be doing less work in reconstituting the AmBisome for patients only once and not for 7 days. Task shifting in reconstituting the AmBisome to pharmacists or other trained staff would lessen the nursing burden.

While there are some challenges with AmBisome, we have more than a decade of experience in using amphotericin B deoxycholate which informs our overall perspective ^12– 14^. Deoxycholate requires a shorter daily reconstitution time of ~5 minutes; however, this adds up over 7–14 days to be 35–70 minutes of total nursing time, more than with the single AmBisome infusion. When using peripheral intravenous cannula sites, thrombophlebitis frequently occurs, and we generally rotate intravenous sites every three days, which adds additional nursing time and supplies. Deoxycholate infusions generally take four hours or more. If the infusion rates are set too quickly, infusion reactions intensify. When reactions occur, additional medications may need to be given, requiring additional nursing care. With amphotericin infusion, this monitoring of patients requires additional nursing time ideally, thus we estimate approximately 6 hours of nursing time is saved for 1 infusion versus 7 daily infusions. The cumulative toxicities of amphotericin B deoxycholate are well known where hypokalaemia, anaemia, and kidney injury are all risk factors for death ^20– 22^. Thus, in comparison to deoxycholate formulations, the single-dose liposomal amphotericin therapy is very favourable from a nursing perspective.

The other major challenge we realised with using AmBisome was the high cost. Amphotericin B deoxycholate costs US$14.88 (not including cold-chain shipping) per 50mg dose which is given in 7 daily doses ($104 in total). However, AmBisome costs over US$300 for a single 50mg vial, which would cost over US$12,000 for a traditional two-week course at 3mg/kg. Therefore, even with evidence of safety and efficacy, it may be challenging to convince Ministries of Health in low- and middle-income countries to adopt AmBisome when there is a cheaper option. However, recently, Gilead Sciences agreed to preferential pricing in low- and middle-income countries ^23^. AmBisome is also currently available within a programme funded by Unitaid who, in partnership with the Clinton Health Access Initiative (CHAI), are providing antifungal medication to countries with a high prevalence of cryptococcal meningitis ^24^. However, this program may close soon. Data from the AMBITION-cm trial showed that the single, high-dose AmBisome regimen was cost-effective in comparison to the current WHO-recommended standard of care at $143 per life-year saved. This effect is likely to be greater in real-world implementation and ongoing advocacy is needed to continue the availability of AmBisome for patients in resource-limited settings where the burden is highest.

## Conclusion

In conclusion, liposomal amphotericin B is a better alternative to amphotericin B deoxycholate with less toxicity, nosocomial infections, reduced hospital stay, and the potential for lower costs of hospitalization for both the patient and the healthcare system given the single, 10 mg/kg regimen. Despite the perceived high cost of liposomal amphotericin and the tedious reconstitution process requiring training and adequate manpower, we believe that single-dose AmBisome has enough advantages over the deoxycholate formulation to compel Ministries of Health to consider procuring AmBisome as the drug of choice for the management of HIV-associated cryptococcal meningitis. Liposomal amphotericin is already included in the WHO Model List of Essential Medicines ^25^. The rollout will require training to facilitate widespread implementation, and we hope that this paper will serve to facilitate this training for nurses who will be using liposomal amphotericin. However, more clinical trials to explore the utility of single-dose 10mg/kg AmBisome in other invasive fungal infections globally are warranted.

## Ethics and consent

The study was conducted in compliance with the approved protocol, the Declaration of Helsinki 2008, the principles of Good Clinical Practice and applicable national regulations. The protocol was approved by the London School of Hygiene and Tropical Medicine Research Ethics Committee (on 09/06/2017, ref: 14322) and Mulago Hospital Research and Ethics Committee (MHREC 1297). All the participants provided written informed consent.

## Data Availability

All data underlying the results are available as part of the article and more information about Ambition-cm trial can be found at https://www.nejm.org/doi/full/10.1056/NEJMoa2111904#ap4 and the results of the main clinical trial can be found in Jarvis *et al.*, 2022 ^17^.
