# Supplementary material for: Using single-dose liposomal amphotericin B for cryptococcal meningitis induction therapy: nurse pearls and practical perspectives
Source: Wellcome Open Res. 2024 Oct 17;9:253. Originally published 2024 May 15. [Version 2] doi: 10.12688/wellcomeopenres.21450.2 (PMC11462120; doi:10.12688/wellcomeopenres.21450.2)
Supplement: Peer review history

# Reviewer report: Reviewer response for version 2

**DOI:** 10.21956/wellcomeopenres.25658.r115649

**Published:** 2024-12-17

**Authors:** Jiahui Cheng

**Affiliations:** 1 Karolinska Institute, Solna, Stockholm, Sweden

This study demonstrates a practical situation of single-dose liposomal amphotericin B in source-limited area from nursing perspectives nested under the large scale and well-organized RCT. The authors illustrate the overall advantage of single-dose AmBisome compared with conventional amphotericin B deoxycholate, by describing the reduced time of preparation and administration, non-inferior efficacy, and fewer drug-related adverse events which shorten the hospital stay. Thus, the study clearly provides a favorable attitude towards AmBisome when initially treating HIV-infected cryptococcal meningitis, to lessen the overall burden of medical system.

 As an observational study based on RCT, this study is well-designed, conducted reliably, and clarified clearly.

 Here is a minor suggestion. From my point of view, most results are presented as a descriptive manner. I suggest more **detailed and comparative numerical data **being provided in Results or Discussion part to make the conclusion more solid and convincing. For instance, the reduced percentage of intravenous cannulation, the lower prevalence of secondary bacteraemia, the fewer median days of hospital stay (and so on if applicable) in patients receiving AmBisome compared with control arm. Even though some of the numbers are cited from other papers, they provide direct comparisons to show how much effort AmBisome has made to shorten nursing time and subsequently reduce nursing burden.

 In general, this is a well written original article that provides valuable experience in clinical practice regarding HIV-infected patients with cryptococcal meningitis, especially in source-limited area.

Is the work clearly and accurately presented and does it cite the current literature?

Yes

If applicable, is the statistical analysis and its interpretation appropriate?

Not applicable

Are all the source data underlying the results available to ensure full reproducibility?

No source data required

Is the study design appropriate and is the work technically sound?

Yes

Are the conclusions drawn adequately supported by the results?

Yes

Are sufficient details of methods and analysis provided to allow replication by others?

Partly

Reviewer Expertise:

infectious diseases, cryptococcal meningitis

I confirm that I have read this submission and believe that I have an appropriate level of expertise to confirm that it is of an acceptable scientific standard.

# Reviewer report: Reviewer response for version 2

**DOI:** 10.21956/wellcomeopenres.25658.r109083

**Published:** 2024-11-27

**Authors:** Ajay Rangaraj

**Affiliations:** 1 Department of HIV, World Health Organisation, Geneva, Switzerland

The methods need to include further clarity on how the nurses were trained, what training materials etc. as the study is on nurse pearls - so the trial methodology a little less relevant here. 

 Also in terms of consent for participation etc., for nurses needs to be specified - only information on trial participants has been shared. 

 Also was there a quality of training assessment - e.g., post-assessment?

Is the work clearly and accurately presented and does it cite the current literature?

Yes

If applicable, is the statistical analysis and its interpretation appropriate?

Not applicable

Are all the source data underlying the results available to ensure full reproducibility?

Yes

Is the study design appropriate and is the work technically sound?

Partly

Are the conclusions drawn adequately supported by the results?

Yes

Are sufficient details of methods and analysis provided to allow replication by others?

Partly

Reviewer Expertise:

HIV/AHD/Fungal infections/Cervical Cancer

I confirm that I have read this submission and believe that I have an appropriate level of expertise to confirm that it is of an acceptable scientific standard, however I have significant reservations, as outlined above.

# Reviewer report: Reviewer response for version 2

**DOI:** 10.21956/wellcomeopenres.25658.r109081

**Published:** 2024-11-27

**Authors:** Vincent Ganu

**Affiliations:** 1 Korle-Bu Teaching Hospital, Accra, Ghana

I think the presentation of this article in the format of an original research is problematic. It seems to me it is just a descriptive  narrative (which requires lots of improvement in the write up for clarity and clearer flow) of what nurses did and therefore may best be presented as a short communication or letter to the editor or an editorial .

**Introduction:**

 The study seeks to talk about nurses’ perspective or experience with amphotericin. Agreed that, liposomal amphotericin is new, the introduction lacks literature with evidence of what the nursing experience has been with amphotericin B doxycholate, the challenges or advantages and disadvantages, difficulties in handling the deoxycholate (e.g, dosing calculation, mixing, administration etc) and potential effects on the patients. What are the existing gaps in the nursing experience with amphotericin B thus necessitating this study on nursing experience on liposomal amphotericin B? The introduction should be based on the subject matter. The study is about nursing experience or perspectives and therefore that has to be the main focus of the entire work being presented here.

 Although the literature presented here on Amphotericin is ok, it is not enough to address the subject matter for the reading audience.

**Methods:**

 The study design stated is not the study design for the subject matter in this paper. Kindly note that there is a bigger clinical trial which is on-going and the manuscript here is looking at nursing experience which is a subset of the bigger clinical trial. And therefore the design should be different. Was it a cross-sectional study that used a qualitative only approach, or a quantitative only approach, or a mixed method approach? The authors need to think through this and decide on the design used.

 There are no statements on the procedures that were conducted to achieve each of the four aims or objectives described in the last paragraph of the introduction section. How was experience assessed? How was the preparation and administration assessed? How was the required time assessed for the daily amphotericin and single-dose liposomal assessed?

**Results:**

 Most, if not all, of the content of the results are not results but rather procedures which should be under the methods section.

 Please view the additional review comments in the file found here.

Is the work clearly and accurately presented and does it cite the current literature?

No

If applicable, is the statistical analysis and its interpretation appropriate?

No

Are all the source data underlying the results available to ensure full reproducibility?

No

Is the study design appropriate and is the work technically sound?

No

Are the conclusions drawn adequately supported by the results?

No

Are sufficient details of methods and analysis provided to allow replication by others?

No

Reviewer Expertise:

Implementation research, HIV and opportunistic infections

I confirm that I have read this submission and believe that I have an appropriate level of expertise to state that I do not consider it to be of an acceptable scientific standard, for reasons outlined above.

# Reviewer report: Reviewer response for version 2

**DOI:** 10.21956/wellcomeopenres.25658.r109076

**Published:** 2024-11-19

**Authors:** Nagarathna Chandrashekar

**Affiliations:** 1 Department of Neuromicrobiology, National Institute of Mental Health and Neuro Sciences, Bangalore, India

Manuscript is very well written highlighting the dedication, diligence and time required in the preparation of the drug by the nurses before its administration. The article describes the cost effectiveness ,reduced patient care time and health care associated risks involved in the process. L-AMB has advantages from the nurses perspective as well.

Is the work clearly and accurately presented and does it cite the current literature?

Yes

If applicable, is the statistical analysis and its interpretation appropriate?

Not applicable

Are all the source data underlying the results available to ensure full reproducibility?

Yes

Is the study design appropriate and is the work technically sound?

Yes

Are the conclusions drawn adequately supported by the results?

Yes

Are sufficient details of methods and analysis provided to allow replication by others?

Yes

Reviewer Expertise:

Infections of the Central Nervous System.Cryptococcal infections and other fungal infections of the central Nervous System

I confirm that I have read this submission and believe that I have an appropriate level of expertise to confirm that it is of an acceptable scientific standard.

## References

[1] : AmBisome® Formulations for Pediatrics: Stability, Cytotoxicity, and Cost-Effectiveness Studies. *Pharmaceutics* .2024;16(4) : 10.3390/pharmaceutics16040466 10.3390/pharmaceutics16040466 38675127 PMC11054559

# Reviewer report: Reviewer response for version 1

**DOI:** 10.21956/wellcomeopenres.23718.r102529

**Published:** 2024-10-08

**Authors:** Carolina Firacative

**Affiliations:** 1 Studies in Translational Microbiology and Emerging Diseases (MICROS) Research Group, Universidad del Rosario, Bogota, Colombia

The study by Ndyetukira et al. shows the experience of two centers in Uganda, a country with high burden of CM, in processing, preparing and administering single dose L-AmB, highlighting the various advantages of this formulation over the use of amphotericin B deoxycholate. The manuscript is well written and the conclusions are appropriate.

**Some minor points could be addressed before the manuscript is ready for indexing:**

- There is already a study -perhaps more- on the use of L-AmB to treat other fungal infections. Perhaps the authors can include this in the discussion. Pasqualotto et al Clin Infect Dis. 2023;13;77(8):1126-11321.
- Harrison et al, Clin Infect Dis, 2023;76(5):944-949, made an estimation on the advantages, in terms of costs, that AMBITION has. This could be mentioned in the discussion.
- In “Study design and setting”, *Cryptococcus* must be written in italics and capital C. Define CSF as it is used here for the first time.
- In “Study participants” use CSF instead of cerebrospinal fluid. How many patients were involved?
- Results: define IV.

Is the work clearly and accurately presented and does it cite the current literature?

Yes

If applicable, is the statistical analysis and its interpretation appropriate?

Not applicable

Are all the source data underlying the results available to ensure full reproducibility?

Yes

Is the study design appropriate and is the work technically sound?

Yes

Are the conclusions drawn adequately supported by the results?

Yes

Are sufficient details of methods and analysis provided to allow replication by others?

Yes

Reviewer Expertise:

Medical Mycology; Epidemiology

I confirm that I have read this submission and believe that I have an appropriate level of expertise to confirm that it is of an acceptable scientific standard.

## References

[1] : Single High Dose of Liposomal Amphotericin B in Human Immunodeficiency Virus/AIDS-Related Disseminated Histoplasmosis: A Randomized Trial. *Clin Infect Dis* .2023;77(8) : 10.1093/cid/ciad313 1126-1132 10.1093/cid/ciad313 37232940 PMC10573726

[2] : How Applicable Is the Single-Dose AMBITION Regimen for Human Immunodeficiency Virus-Associated Cryptococcal Meningitis to High-Income Settings?. *Clin Infect Dis* .2023;76(5) : 10.1093/cid/ciac792 944-949 10.1093/cid/ciac792 36166405 PMC9989135

# Reviewer report: Reviewer response for version 1

**DOI:** 10.21956/wellcomeopenres.23718.r91181

**Published:** 2024-08-12

**Authors:** Olivier Paccoud

**Affiliations:** 1 Department of Infectious Diseases and Tropical Medicine, University Paris Cité, Paris, Île-de-France, France

In this well written manuscript, Ndyetukira et al describe the nursing experience of using the single dose liposomal amphotericin B regimen in two referral centres from the AMBITION-cm trial. The authors present various benefits of the single-dose regimen, such as reduced overall preparation time and lower rate of infusion related complications, making a compelling case that this regimen is preferable from a nursing perspective in Uganda.  The authors also provide a helpful guide for AmBisome reconstitution. Overall, this manuscript highlights an important yet unfortunately sometimes overlooked aspect of patient care in the evaluation of new treatment regimen.

**Please find below some minor comments:**

- Please consider providing a little bit more context on the in-hospital management of patients with CM usually cared for in the two referral centres in Kampala and Mbarare, such as rough estimates of hospital beds, number of patients per ward, size of the nursing staff etc.. so that the reader may have a better overview of the hospital setting in Uganda.
- One other important aspect that can also be mentioned in the discussion regarding nursing time and overall strain on hospital resources is the potential reduction in length of hospital stays associated with single dose AMBITION-cm regimen compared to 7 or 14 day treatment regimen. As stated by Lawrence *et al.* (2022 ^1^ ) in their cost-effectiveness study, some of the actual benefits of using the AMBITION-cm trial regimen may not have been apparent in the trial setting as patients had to stay hospitalised for at least 7 days.
- It may be useful to remind the reader that AmBisome should never be reconstituted with saline fluid (only dextrose solutions), as this leads to precipitation.
- To clarify, please specify that the drug reactions mentioned in the results section are from the AMBITION-cm trial results and not only in the two sites of this study.
- In the discussion, consider adding a mention of Lawrence *et al. *(2022 ^2^ ) qualitative study on the acceptability of the AMBITION-cm treatment regimen which includes quotes from participants and nurses and emphasises some of the benefits of the treatment regimen discussed in this manuscript.

Is the work clearly and accurately presented and does it cite the current literature?

Yes

If applicable, is the statistical analysis and its interpretation appropriate?

Not applicable

Are all the source data underlying the results available to ensure full reproducibility?

No source data required

Is the study design appropriate and is the work technically sound?

Yes

Are the conclusions drawn adequately supported by the results?

Yes

Are sufficient details of methods and analysis provided to allow replication by others?

Yes

Reviewer Expertise:

invasive fungal infections

I confirm that I have read this submission and believe that I have an appropriate level of expertise to confirm that it is of an acceptable scientific standard.

## References

[1] : Cost-effectiveness of single, high-dose, liposomal amphotericin regimen for HIV-associated cryptococcal meningitis in five countries in sub-Saharan Africa: an economic analysis of the AMBITION-cm trial. *Lancet Glob Health* .2022;10(12) : 10.1016/S2214-109X(22)00450-8 e1845-e1854 10.1016/S2214-109X(22)00450-8 36400090 PMC10009915

[2] : The acceptability of the AMBITION-cm treatment regimen for HIV-associated cryptococcal meningitis: Findings from a qualitative methods study of participants and researchers in Botswana and Uganda. *PLoS Negl Trop Dis* .2022;16(10) : 10.1371/journal.pntd.0010825 e0010825 10.1371/journal.pntd.0010825 36279300 PMC9632910
